# MYB Gene Family in Potato (*Solanum tuberosum* L.): Genome-Wide Identification of Hormone-Responsive Reveals Their Potential Functions in Growth and Development

**DOI:** 10.3390/ijms20194847

**Published:** 2019-09-29

**Authors:** Wenjun Sun, Zhaotang Ma, Hui Chen, Moyang Liu

**Affiliations:** 1 School of Agriculture and Biology, Shanghai Jiao Tong University, Shanghai 200240, China; sunnan82475@163.com (W.S.); WaohUncle_Ma@163.com (Z.M.); 2 College of Life Science, Sichuan Agricultural University, Ya’an 625014, China; chenhui@sicau.edu.cn

**Keywords:** hormone or stress responses, potato genome, *StMYB*, expression patterns

## Abstract

As an important nongrain crop, the growth and yield of potato (*Solanum tuberosum* L.) is often affected by an unfavorable external environment in the process of cultivation. The MYB family is one of the largest and most important gene families, participating in the regulation of plant growth and development and response to abiotic stresses. Several *MYB* genes in potato that regulate anthocyanin synthesis and participate in abiotic stress responses have been identified. To identify all *Solanum tuberosum* L. *MYB* (*StMYB*) genes involved in hormone or stress responses to potentially regulate potato growth and development, we identified the MYB gene family at the genome-wide level. In this work, 158 *StMYB* genes were found in the potato genome. According to the amino acid sequence of the MYB domain and gene structure, the *StMYB* genes were divided into R2R3-MYB and R1R2R3-MYB families, and the R2R3-MYB family was divided into 20 subgroups (SGs). The expression of 21 *StMYB* genes from different SGs in roots, stems, leaves, flowers, shoots, stolons, young tubers, and mature tubers was determined by quantitative real-time polymerase chain reaction (qRT-PCR). The expression patterns of *StMYB* genes in potatoes treated with abscisic acid (ABA), indole-3-acetic acid (IAA), gibberellin acid 3 (GA3), NaCl, mannitol, and heat were also measured. We have identified several potential candidate genes that regulate the synthesis of potato flavonoids or participate in hormone or stress responses. This work provides a comprehensive understanding of the MYB family in potato and will lay a foundation for the future investigation of the potential functions of *StMYB* genes in the growth and development of potato.

## 1. Introduction

The abiotic stresses that plants encounter in their growth process seriously affect their growth and productivity. At every moment of a plant’s life, thousands of genes are expressed in a coordinated manner to regulate its growth and development and help it resist adverse external environments. Identifying the key genes involved in plant responses to abiotic stress and exploring their molecular mechanisms can lay a foundation for the breeding of crops resistant to abiotic stress [1,2]. Transcription factors (TFs) play a crucial role in plant growth and development through self-regulation and regulation of downstream target gene expression [3]. TFs can have a DNA-binding domain, transcriptional activation domain, and dimerization domain and are involved in physiological and biochemical processes that activate or inhibit transcription in response to endogenous and exogenous stimuli. The MYB family is the largest and most functional TF family in plants, animals, and fungi [4]. The imperfect repeat (R) is the basic unit of the DNA-binding domain of MYB proteins and consists of approximately 52 amino acid residues. Each repeat has a helix–turn–helix (HTH) structure motif, which is composed of three conserved tryptophan residues (W) with an interval of 18–19 amino acids. According to the number of Rs contained in each MYB protein, the MYB proteins can be divided into 4R-MYB, R2R3-MYB, R1R2R3-MYB (3R-MYB), and MYB-related proteins [5,6]. 4R-MYB is the smallest class. Each gene in this class contains four R1/R2 repeats, and little is known about these proteins in plants. The second class of the MYB family contains three continuous repeats (R1R2R3-MYB), and 3R-MYB proteins have been found in most eukaryotic genomes, playing a role in cell cycle control [7]. The third class of the MYB family, called MYB-related, contains a single or partial MYB repeat [8,9,10]. Among these four classes, R2R3-MYB is the largest in plants, and the genes of this family may have evolved from the loss of the R1 repeat in the R1R2R3-MYB gene [11]. However, it has also been suggested that the 3R-MYB gene evolved from the R2R3-MYB gene, which acquired the R1 repeat [12]. There is a highly conserved MYB domain in the structure of R2R3-MYB TFs, and their C-termini are highly variable. R2R3-MYB usually determines the interaction between proteins [6,13,14].

The R2R3-MYB family is widely involved in various physiological activities of plants, such as responding to environmental factors and plant hormones, participating in the regulation of primary and secondary metabolism, regulating cell cycle and cell differentiation, and leaf morphogenesis [15,16]. According to the conserved domains of *MYB* genes and their functions, the R2R3-MYB family in *Arabidopsis thaliana* (L.) Heynh. is divided into 23 subgroups (SGs) [5]. Each subgroup plays an important role in different physiological and biochemical activities. Members of SG 7 have a conserved GRTxRSxMK motif, which can positively regulate the biosynthesis of flavonoids. *AtMYB11*, *AtMYB12*, and *AtMYB111* in SG 7 were found to be involved in the regulation of flavonoid biosynthesis in *Arabidopsis thaliana* (L.) Heynh. [17]. Members of SG 5 have a conserved DExWRLxxT motif, and members of SG 6 have a conserved KPRPRFb motif, which can form complexes with members of the WD40 and bHLH families to participate in the biosynthesis of proanthocyanidins and anthocyanins, respectively. It has been found that *AtMYB75*, *AtMYB90*, *AtMYB113*, and *AtMYB114* in SG 6 can regulate anthocyanin biosynthesis, while *AtMYB123* in SG 5 can regulate proanthocyanidin synthesis in the seed coat of *Arabidopsis thaliana* (L.) Heynh. [18,19].

Studies have shown that R2R3-MYB family genes also play an important role in the plant responses to abiotic stress, besides participating in various physiological activities. Several *MYB* genes involved in stress response have been identified in wheat, among which *TaMYB1* can respond to abiotic stress and ABA treatment, *TaMYB2A* in transgenic *Arabidopsis thaliana* (L.) Heynh. showed a variety of abiotic stress tolerances, while *TaMYB33* can reconstruct the osmotic balance of transgenic plants, thereby improving their salt tolerance and drought tolerance [20,21,22]. Owing to the powerful function of *MYB* genes, an increasing number of *MYB* genes have been identified, and the MYB gene family has been identified from many dicotyledonous and monocotyledonous plants, including *Arabidopsis thaliana* (L.) Heynh. (197 members) [23], *Cucumis sativus* L. (55 members) [6], *Pyrus bretschneideri* Rehd. (184 members) [24], *Beta vulgaris* L. (70 members) [25], *Gossypium hirsutum* Linn. (524 members) [26], *Oryza sativa* L. (155 members) [23], and *Zea mays* Linn. (158 members) [27] at the genome-wide level.

In the potato cultivation process, abiotic stresses, including drought and high salt, often harm potato growth and lead to a decline in yield. Therefore, it is urgent to explore potato stress resistance genes for potato breeding and production. At present, some *MYB* genes involved in stress response have been identified in potato. For example, *StMYB1R-1* is involved in activating drought-related genes and improves potato drought tolerance by regulating water loss [28]. In addition, studies have shown that in transgenic potato, the increased expression of *IbMYB1* affects secondary metabolism, thereby improving potato tolerance to stress [29]. Li et al. previously identified the MYB gene family from potato, and analyzed the chromosome location, syntenic, and the role of *MYB* genes in potato development and stress response [30]. In this study, 158 *MYB* genes were identified from the potato genome by different identification methods, and genes related to growth, development, and stress response were excavated. Their motif composition, intron and exon distribution, and tandem duplication and segmental duplication events were comprehensively analyzed. Their evolutionary relationships with dicotyledonous *Arabidopsis thaliana* (L.) Heynh., *Fagopyrum tataricum* Gaertn., *Manihot esculenta* Crantz, *Nicotiana attenuate*, *Solanum lycopersicum*, *Vitis vinifera* L., and monocotyledonous *Oryza sativa* L. were compared. At the same time, we measured the expression levels of *StMYB* genes in roots, stems, leaves, flowers, shoots, stolons, young tubers, and mature tubers. More importantly, by determining the expression patterns of *StMYB* genes under ABA, IAA, GA3, NaCl, mannitol, and high temperature treatments, we identified some *StMYB* genes that may potentially play a vital role in potato growth and hormone stress resistance. This study can provide a meaningful reference for potato breeding improvement.

## 2. Results

### 2.1. Identification of the MYB Gene Family in Potato

We excavated 158 *MYB* genes from the potato genome and named them *StMYB1* to *StMYB158* according to their distribution on the chromosomes (Table S1). The coding sequence lengths, molecular weights (MWs), subcellular localizations, and isoelectric points (PIs) of these genes are shown in Table S1. StMYB26 had the smallest amino acid sequence of 53 aa, while StMYB146 had the largest amino acid sequence of 1021 aa. The MWs of these StMYB proteins ranged from 5.89 kDa (StMYB26) to 113.39 kDa (StMYB146), and their PIs ranged from 4.59 (StMYB78) to 10.26 (StMYB10). StMYB140 was located in chloroplasts; StMYB10, StMYB26, StMYB37, and StMYB138 were located in mitochondria; and other StMYB proteins were located in the nucleus (Table S1).

### 2.2. Phylogenetic Tree Analysis of MYB Genes in Potato and Arabidopsis thaliana (L.) Heynh

**Figure 1:**
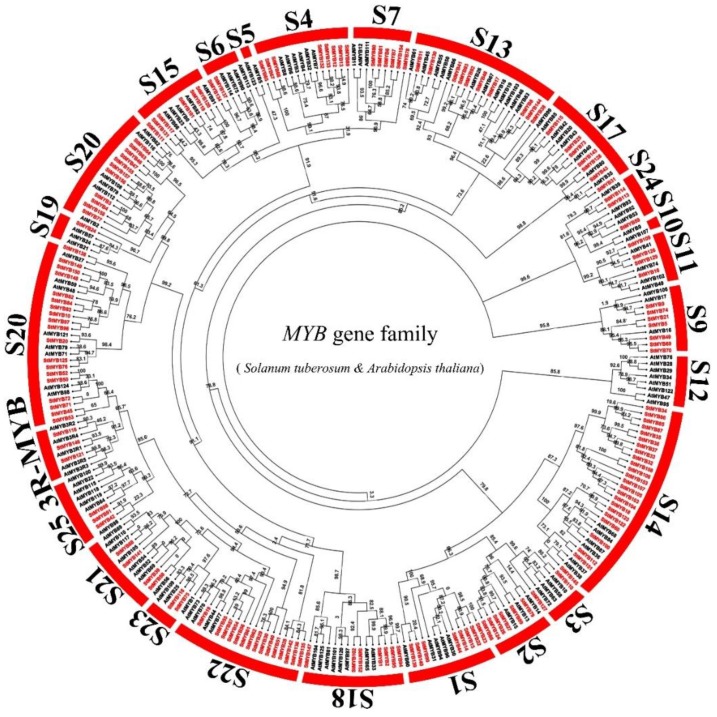
Unrooted phylogenetic tree representing the relationships among 158 *MYB* genes of potato and *Arabidopsis thaliana* (L.) Heynh. with 1000 bootstrap replicates by the maximum likelihood (ML) method. *MYB* genes from potato and *Arabidopsis thaliana* (L.) Heynh. are classified into S1, S2, S3, S4, S5, S6, S7, S9, S10, S11, S12, S13, S14, S15, S17, S18, S19, S20, S21, S22, S23, S24, and S25. The genes in potato are marked in red, while those in *Arabidopsis thaliana* (L.) Heynh. are marked in black.

We constructed a maximum likelihood (ML) phylogenetic tree with 158 StMYB proteins and 128 MYB proteins from *Arabidopsis thaliana* (L.) Heynh. using Mega 7.0. Five of 128 *MYB* genes in *Arabidopsis thaliana* (L.) Heynh. belonged to the 3R-MYB subfamily, and the others belonged to the R2R3-MYB subfamily. The R2R3-MYB subfamily was further divided into S 1, S 2, S 3, S 4, S 5, S 6, S 7, S 9, S 10, S 11, S 12, S 13, S 14, S 15, S 17, S 18, S 19, S 20, S 21, S 22, S 23, S 24, and S 25. From the phylogenetic tree, we can see that three *StMYB* genes belonged to the 3R-MYB subfamily (*StMYB116*, *StMYB121,* and *StMYB146*), while 155 R2R3-MYB subfamily genes belonged to S 1 (8 members), S 2 (6 members), S 3 (2 members), S 4 (8 members), S 6 (2 members), S 7 (6 members), S 9 (7 members), S 11 (4 members), S 13 (10 members), S 14 (24 members), S 15 (6 members), S 17 (7 members), S 18 (7 members), S 19 (1 member), S 20 (30 members), S 21 (4 members), S 22 (15 members), S 23 (2 members), S 24 (3 members), and S 25 (3 members) subgroups (Figure 1). There were no *StMYB* genes in S 5, S 10, or S 12.

### 2.3. Gene Structure, Sequence Composition, and Promoter Cis-Acting Element Analysis

**Figure 2:**
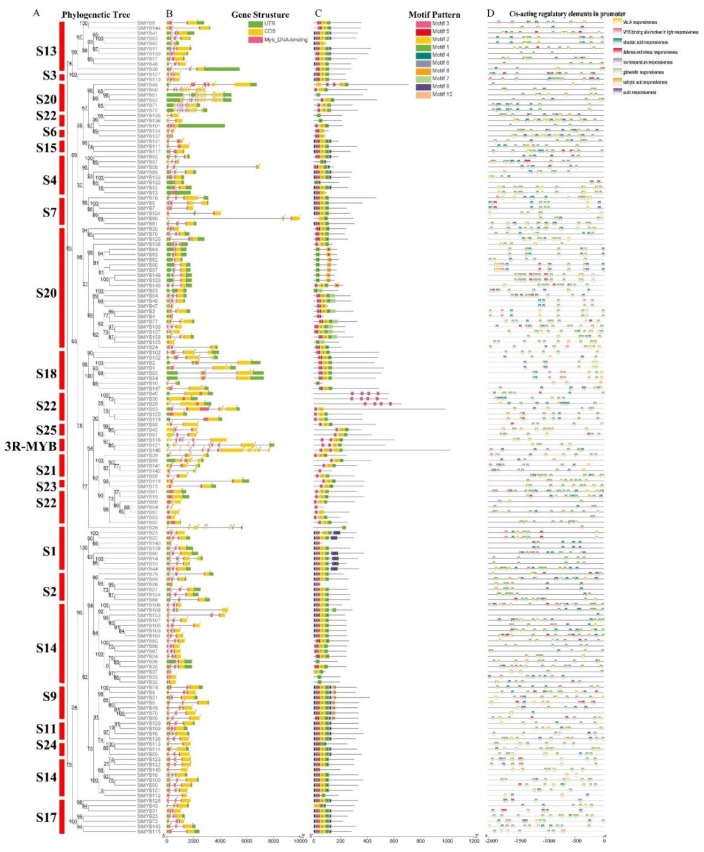
Phylogenetic relationships, gene structures, architectures of the conserved protein motifs, and cis-acting elements analysis of the *MYB* genes from potato. (**A**) The phylogenetic tree was constructed based on the full-length sequences of potato MYB proteins using Geneious R11 software. (**B**) Exon-intron structures of potato *MYB* genes. Blue boxes indicate untranslated 5′- and 3′-regions, and black lines indicate introns. The number indicates the phases of the corresponding introns. (**C**) The motif compositions of the potato MYB proteins. The motifs, numbered 1–10, are displayed in different colored boxes. The sequence information for each motif is provided in Table S2. The protein length can be estimated using the scale at the bottom. (**D**) The cis-acting elements analysis of *StMYB* genes promoters. Blocks of different colors represent light responsiveness elements, low temperature responsiveness elements, salicylic acid responsiveness elements, abscisic acid responsiveness elements, MeJA responsiveness elements, auxin responsiveness elements, gibberellin responsiveness elements, and defense and stress responsiveness elements.

The phylogenetic tree was constructed from the amino acid sequences of 158 *StMYB* genes, and the R2R3-MYB family genes were still divided into 20 subgroups (Figure 2A). Figure 2B shows that all 158 *StMYB* genes contained a Myb DNA-binding domain and 1 to 10 introns. Some *StMYB* genes had a large number of introns, including *StMYB116* (6 introns), *StMYB121* (7 introns), and *StMYB146* (10 introns) from the 3R-MYB family and *StMYB45* (5 introns), *StMYB50* (9 introns), *StMYB51* (7 introns), *StMYB52* (10 introns), *StMYB71* (7 introns), and *StMYB72* (8 introns) in S 20. The other *StMYB* genes had 1 to 3 introns, and the number of introns of *StMYB* genes in the same subgroup was generally similar.

By detecting the motif composition of StMYB proteins characteristic regions, it was found that StMYB51, StMYB135, StMYB136, StMYB10, StMYB28, StMYB36, StMYB33, and StMYB32 only contain motif 1 (Figure 2C). Proteins containing only motif 3 included StMYB40, StMYB30, StMYB29, StMYB142, and StMYB54. StMYB67 and StMYB132 only contained motifs 1 and 6; StMYB80 only contained motifs 1 and 2; StMYB4 only contained motifs 3 and 5; StMYB155 and StMYB63 only contained motifs 1 and 7; StMYB53 and StMYB57 only contained motifs 2 and 3; and proteins containing only motifs 1 and 8 included StMYB84, StMYB83, StMYB82, StMYB96, StMYB97, StMYB148, and StMYB150. Almost all the other proteins contained motifs 2 and 3. Only the proteins of S 1 contained motif 9, while those of S 9 contained motif 10. In conclusion, most StMYB proteins from the same subgroup had similar motif compositions (Figure 2C).

The promoter prediction results showed that the cis-acting elements in the promoter region of *StMYB* genes included MeJA responsiveness elements, MYB-binding sites that were part of light responsiveness elements, ABA responsiveness elements, defense and stress responsiveness elements, low temperature responsiveness elements, gibberellin acid (GA) responsiveness elements, salicylic acid (SA) responsiveness elements, and auxin responsiveness elements (Figure 2D). These cis-acting elements could be classified into three categories, including phytohormones, environmental stress, and photoresponsive elements (Figure 2D). The GA responsiveness elements were most common in the *StMYB* gene promoters, which were present in 149 promoters. There were 113 *StMYB* gene promoters containing ABA responsiveness elements, 87 *StMYB* gene promoters containing MeJA responsiveness elements, 72 *StMYB* gene promoters containing SA responsiveness elements, 49 *StMYB* gene promoters containing defense and stress responsiveness elements, 46 *StMYB* gene promoters containing light responsiveness elements, 41 *StMYB* gene promoters containing low temperature responsiveness elements, and 24 *StMYB* gene promoters containing auxin responsiveness elements. In general, phytohormone responsiveness elements other than auxin responsiveness elements were most common in *StMYB* gene promoters (Figure 2D).

### 2.4. Analysis of Chromosome Distribution, Tandem Gene Duplication, and Segmental Gene Duplication of StMYB Genes

**Figure 3:**
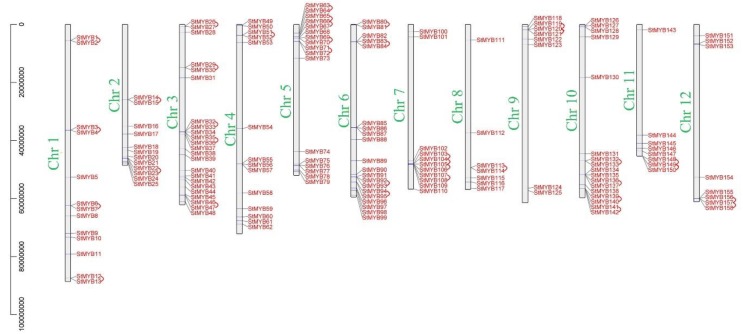
The distribution of *MYB* genes on chromosomes. The chromosome number is indicated to the left of each chromosome.

The 158 *StMYB* genes were unevenly distributed on 12 chromosomes (Figure 3). There were 23 *StMYB* genes on chromosome 3, 20 *StMYB* genes on chromosome 6, 17 *StMYB* genes on chromosome 5 and chromosome 10, 14 *StMYB* genes on chromosome 4, 13 *StMYB* genes on chromosome 1, 12 *StMYB* genes on chromosome 2, 11 *StMYB* genes on chromosome 7, 14 *StMYB* genes on chromosome 9, chromosome 11 and chromosome 12, and 7 *StMYB* genes on chromosome 8 (Figure 3). A total of 36 tandem-duplicated gene pairs were unevenly distributed on 12 chromosomes. There were 5 tandem-duplicated gene pairs on chromosome 3 (*StMYB26* and *StMYB27*, *StMYB29* and *StMYB30*, *StMYB32* and *StMYB33*, *StMYB35* and *StMYB36*, *StMYB46* and *StMYB47*) and chromosome 5 (*StMYB63* and *StMYB64*, *StMYB65* and *StMYB66, StMYB66* and *StMYB67, StMYB69* and *StMYB70, StMYB71* and *StMYB72)*; 4 tandem-duplicated gene pairs on chromosome 1 (*StMYB1* and *StMYB2, StMYB3* and *StMYB4, StMYB6* and *StMYB7, StMYB12* and *StMYB13*), chromosome 6 (*StMYB80* and *StMYB81, StMYB83* and *StMYB84, StMYB92* and *StMYB93, StMYB94* and *StMYB95)*, chromosome 7 (*StMYB103* and *StMYB104, StMYB104* and *StMYB105, StMYB105* and *StMYB106, StMYB107* and *StMYB108*), and chromosome 10 (*StMYB132* and *StMYB133, StMYB136* and *StMYB137, StMYB139* and *StMYB140, StMYB141* and *StMYB142*); 2 tandem-duplicated gene pairs on chromosome 2 (*StMYB14* and *StMYB15, StMYB22* and *StMYB23*), chromosome 9 (*StMYB119* and *StMYB120, StMYB120* and *StMYB121*), chromosome 11 (*StMYB148* and *StMYB149, StMYB149* and *StMYB150*), and chromosome 12 (*StMYB156* and *StMYB157, StMYB157* and *StMYB158*); and only one tandem-duplicated gene pair on chromosome 4 (*StMYB51* and *StMYB52*) and chromosome 8 (*StMYB113* and *StMYB114*) (Figure 3).

**Figure 4:**
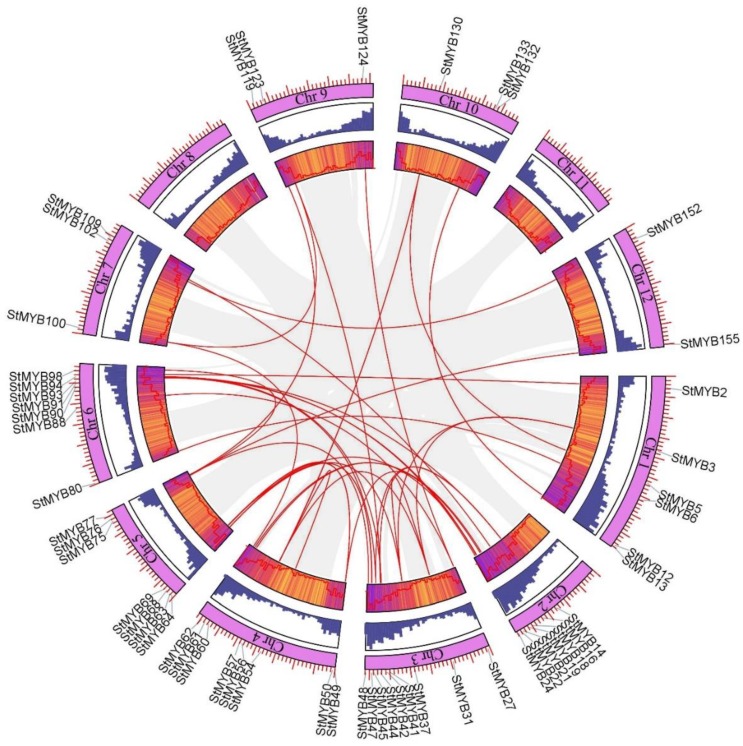
The interchromosomal relationships of the potato *MYB* genes. Colored lines indicate all syntenic blocks in the potato genome.

In addition to tandem-duplicated gene pairs, 41 pairs of segmental-duplicated gene pairs were also found on potato chromosomes (Figure 4). All in all, by analyzing the duplication events in *StMYB* genes, we found that some *StMYB* genes were produced by tandem duplication and segmental duplication, and these gene duplication events may be one of the drivers of the evolution of *StMYB* genes.

### 2.5. Evolutionary Analysis of StMYB Genes and the MYB Genes of Several Different Species

**Figure 5:**
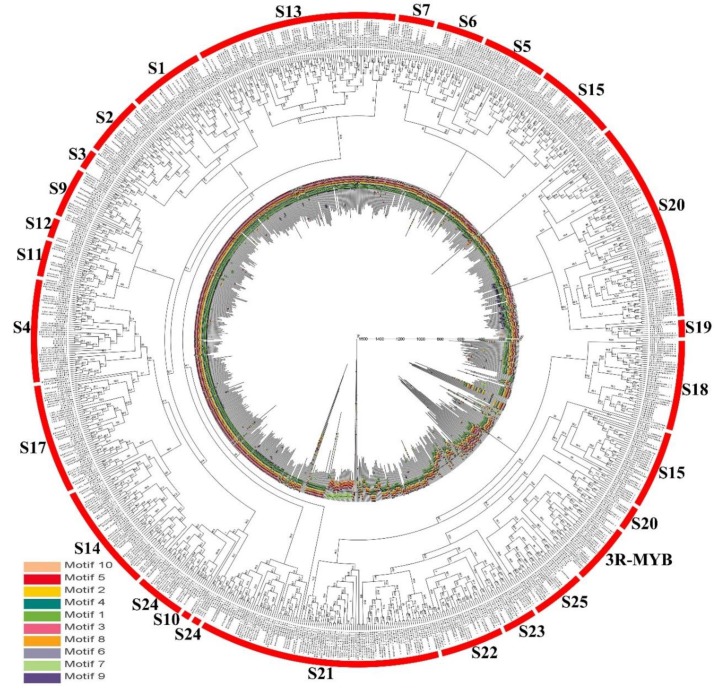
Phylogenetic relationships and motif compositions of the MYB proteins from six different plant species (*Solanum tuberosum* L., *Arabidopsis thaliana* (L.) Heynh., *Solanum lycopersicum*, *Vitis vinifera* L., *Manihot esculenta* Crantz, and *Oryza sativa* L.). *MYB* genes from multiple species are classified into S 1, S 2, S 3, S 4, S 5, S 6, S 7, S 9, S 11, S 12, S 13, S 14, S 15, S 17, S 18, S 19, S 20, S 21, S 22, S 23, S 24, and S 25. Outer layer: An unrooted ML phylogenetic tree constructed using Mega 7.0. Inner layer: Distribution of the conserved motifs in MYB proteins. The differently colored boxes represent different motifs and their positions in each MYB protein sequence.

After exploring the evolutionary relationship of *MYB* genes in potato, we constructed a phylogenetic tree including dicotyledonous potato, *Arabidopsis thaliana* (L.) Heynh., *Solanum lycopersicum*, *Vitis vinifera* L., *Manihot esculenta* Crantz, and monocotyledonous *Oryza sativa* L. to explore the evolutionary relationship of *MYB* genes in different plants (Figure 5). As shown in Figure 5, *MYB* genes in all plants were still divided into the 3R-MYB subfamily and the R2R3-MYB subfamily, while the R2R3-MYB subfamily was further divided into S 1, S 2, S 3, S 4, S 5, S 6, S 7, S 9, S 11, S 12, S 13, S 14, S 15, S 17, S 18, S 19, S 20, S 21, S 22, S 23, S 24, and S 25. There were no *MYB* genes from *Oryza sativa* L. in S 2 or S 3; no *StMYB* genes in S 5; no *MYB* genes from potato, *Solanum lycopersicum,* or *Oryza sativa* L. in S 10; and only *MYB* genes from *Arabidopsis thaliana* (L.) Heynh. and *Manihot esculenta* Crantz in S 12. In addition to these subgroups, other subgroups included *MYB* genes from six plants. Ten motifs were detected in all MYB protein sequences, and most MYB proteins contained motifs 1, 2, 4, and 5, while motifs 6 to 9 only existed in a few MYB proteins. *MYB* genes of the same subgroup, especially those with similar homology, have similar motif compositions and similar positions, which further supports the reliability of phylogenetic trees.

**Figure 6:**
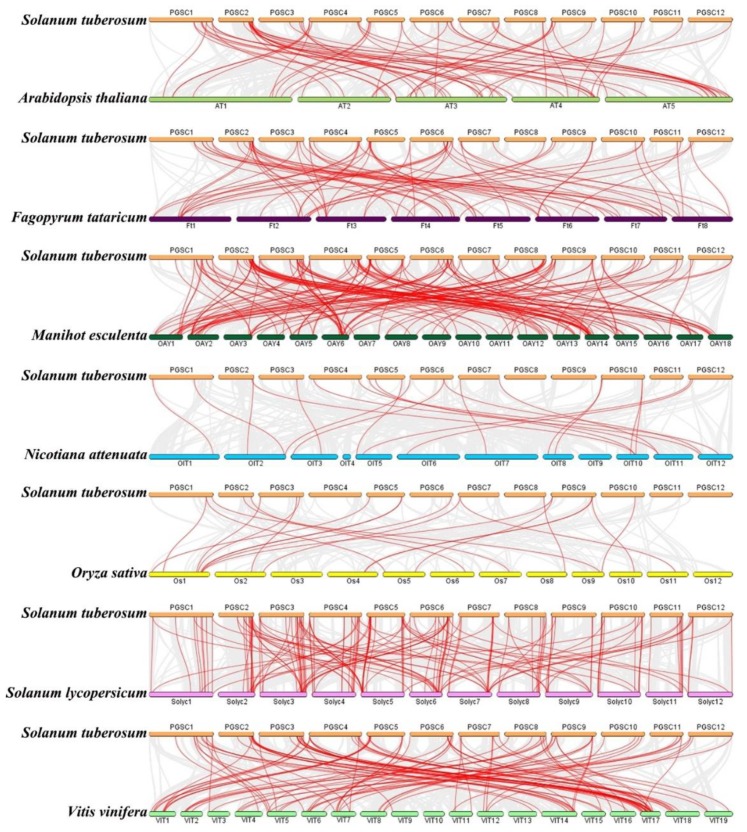
Synteny analyses between the *MYB* genes of potato and seven representative plant species. Gray lines in the background indicate collinear blocks within potato and other plant genomes, while red lines highlight syntenic *MYB* gene pairs.

We further constructed a synteny analysis between *MYB* genes in potato and *MYB* genes in many plants, including dicotyledonous *Arabidopsis thaliana* (L.) Heynh., *Fagopyrum tataricum* Gaertn., *Manihot esculenta* Crantz, *Nicotiana attenuate*, *Solanum lycopersicum*, *Vitis vinifera* L., and monocotyledonous *Oryza sativa* L. (Figure 6). The *StMYB* genes were homologous to genes in other plants, and syntenic conservation was observed among *Solanum lycopersicum* (159 orthologous gene pairs dispersed on all chromosomes), *Manihot esculenta* Crantz (159 orthologous gene pairs dispersed on all chromosomes), *Vitis vinifera* L. (106 orthologous gene pairs dispersed on all chromosomes except chromosome 10), *Fagopyrum tataricum* Gaertn. (70 orthologous gene pairs dispersed on all chromosomes), *Arabidopsis thaliana* (L.) Heynh. (65 orthologous gene pairs dispersed on all chromosomes), *Nicotiana attenuate* (21 orthologous gene pairs dispersed on all chromosomes except chromosome 4 and chromosome 9), and *Oryza sativa* L. (21 orthologous gene pairs dispersed on all chromosomes except chromosome 3 and chromosome 12) (Figure 6). The results of the syntenic analysis showed that *StMYB44* formed homologous gene pairs with *MYB* genes in different plants, including five pairs of homologous gene pairs with *MYB* genes in *Solanum lycopersicum*, three pairs of homologous gene pairs with *MYB* genes in *Manihot esculenta* Crantz, and two pairs of homologous gene pairs with *MYB* genes in *Vitis vinifera* L., which indicated that *StMYB44* may have played a key role in evolution (Table S3).

### 2.6. Expression Patterns of StMYB Genes in Different Plant Tissues

**Figure 7:**
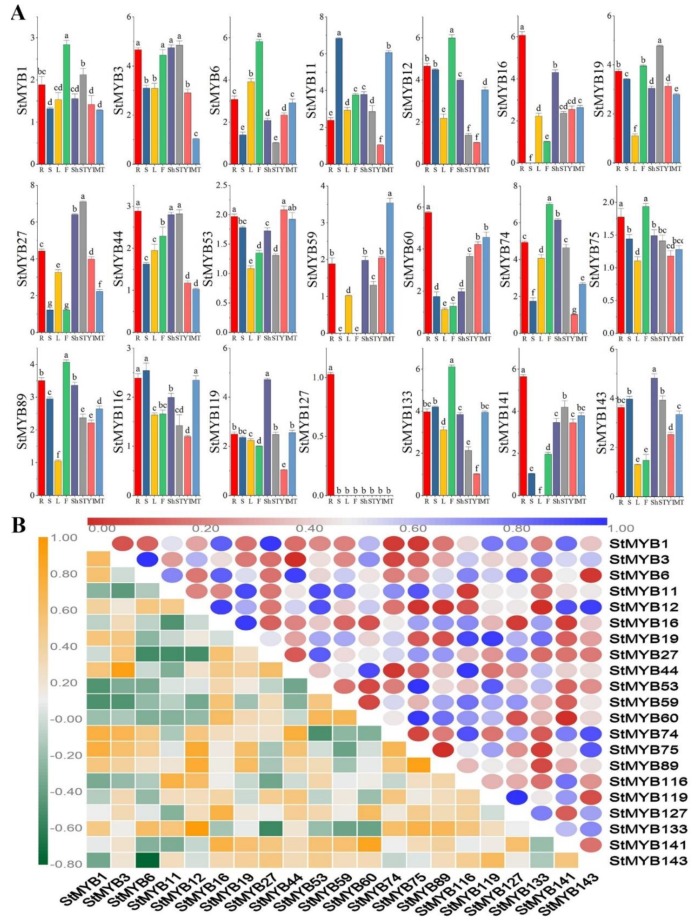
Tissue-specific gene expression of 21 potato *MYB* genes and the correlation between the gene expression patterns of *StMYBs*. (**A**) The expression patterns of 21 potato *MYB* genes in roots, stems, leave, flowers, shoots, stolons, young tuber, and mature tuber were examined by quantitative real-time polymerase chain reaction (qRT-PCR). The expression pattern of each gene was calculated based on the minimum expression of each gene in different tissues. Error bars were obtained from three measurements. Lowercase letter(s) above the bars indicate significant differences (α = 0.05, least significant difference (LSD)) among the treatments. (**B**) Different colors represent the correlation of *StMYB* gene expression patterns in different tissues. Different colors in the lower left corner represent the correlation of *StMYB* gene expression patterns in different tissues, while the different colors in the upper right corner represent the *p*-value of the correlation.

*MYB* genes in different subgroups may perform different functions during the growth and development of potato. To explore their specific functions, we selected a *StMYB* gene from each subgroup and determined their expression levels in roots, stems, leaves, flowers, shoots, stolons, young tubers, and mature tubers by quantitative real-time polymerase chain reaction (qRT-PCR) (Figure 7A). With the exception of *StMYB59*, *StMYB127*, *StMYB16,* and *StMYB141*, other genes were expressed in all tissues, but the expression patterns of each gene were different. Except for the high expression of *StMYB59* in mature tubers, the expression of *StMYB59* was very low in other tissues, but not in stems or flowers. *StMYB127* was only expressed in the root, and the expression level was low. *StMYB16* was not expressed in stems. However, *StMYB141* was not expressed in leaves, and its expression level was low in stems. The expression levels of *StMYB60*, *StMYB44*, *StMYB16*, *StMYB141,* and *StMYB127* were the highest in roots, among which *StMYB127* was a root-specific gene. *StMYB12*, *StMYB6*, *StMYB133*, *StMYB74*, *StMYB1*, *StMYB75,* and *StMYB89* were most expressed in flowers. *StMYB59* and *StMYB53* were most expressed in tubers. *StMYB3*, *StMYB27,* and *StMYB19* were most expressed in stolons. *StMYB119* and *StMYB143* were most expressed in shoots. *StMYB11* and *StMYB116* were most expressed in stems (Figure 7A).

We analyzed the correlation between the expression of these genes in different tissues. It can be seen from Figure 7B that the expression levels of more than half of the genes in different tissues were positively correlated, among which *StMYB3* and *StMYB44*, as well as *StMYB12* and *StMYB133,* were significantly positively correlated.

### 2.7. Expression Analysis of StMYB Genes under Phytohormone and Abiotic Stresses

**Figure 8:**
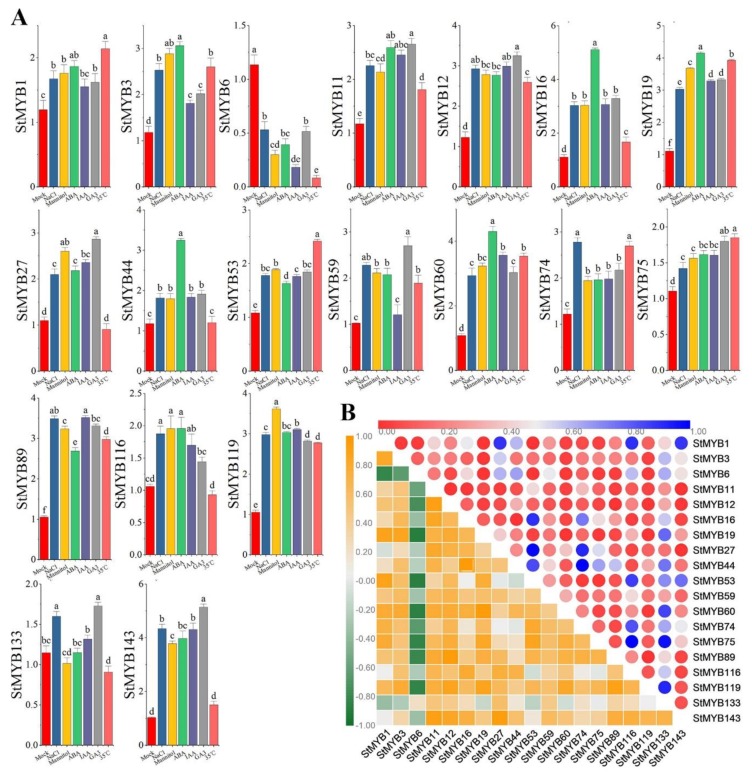
Gene expression of 19 potato *MYB* genes under hormones and abiotic stress and the correlation between the gene expression patterns of *StMYB* genes. (**A**) The expression patterns of 19 potato *MYB* genes under hormones and abiotic stress were examined using a qRT-PCR assay. Error bars were obtained from three measurements. Lowercase letter(s) above the bars indicate significant differences (α = 0.05, LSD) among the treatments. (**B**) Different colors represent the correlation of *StMYB* gene expression patterns under different hormone and abiotic stress treatments. Different colors in the lower left corner represent the correlation of *StMYB* gene expression patterns under different hormone and abiotic stress treatments, while the different colors in the upper right corner represent the *p*-value of the correlation.

The expression levels of *StMYB* genes in ABA, IAA, GA3, high temperature (35 °C), drought, and NaCl were measured by qRT-PCR (Figure 8A). As can be seen from Figure 8A, there were significant expression changes of these genes under different treatments. Except that *StMYB127* and *StMYB141* did not respond to hormone or stress treatments, all genes responded to hormone and abiotic stress treatments, but each gene had different expression patterns under different treatments. The expression levels of *StMYB6* decreased under all stress treatments, *StMYB27* and *StMYB116* decreased under high temperature treatment, *StMYB133* decreased under drought and high-temperature treatment, and the expression levels of other genes increased under hormone and stress treatments. *StMYB60*, *StMYB3*, *StMYB44*, *StMYB16,* and *StMYB19* had the highest expression levels under ABA treatment; *StMYB12*, *StMYB59*, *StMYB133*, *StMYB27*, *StMYB11,* and *StMYB143* had the highest expression levels under GA treatment; *StMYB119* and *StMYB116* had the highest expression levels under drought stress; *StMYB74* had the highest expression level under salt stress; *StMYB1*, *StMYB75,* and *StMYB53* had the highest expression levels under high-temperature treatment; and only *StMYB89* had the highest expression level under IAA treatment.

We analyzed the correlations between the expression levels of these genes under hormone and abiotic stresses. As can be seen from Figure 8B, the expression levels of most genes under hormone and abiotic stresses were positively correlated, among which *StMYB1* was significantly positively correlated with the expression levels of *StMYB75*, *StMYB60*, *StMYB53*, *StMYB19, StMYB6,* and *StMYB3*.

### 2.8. Subcellular Localization of StMYB6 and StMYB19

**Figure 9:**
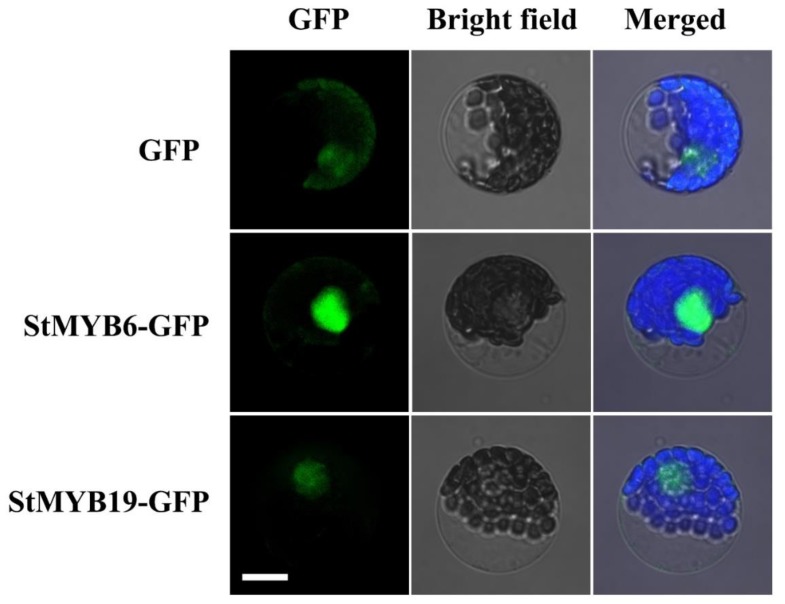
Subcellular localization of *StMYB6* and *StMYB19* in *Arabidopsis thaliana* (L.) Heynh. protoplasts. Enhanced green fluorescent protein (eGFP), StMYB6-eGFP, and StMYB19-eGFP under the control of the CaMV35S promoter separately transiently expressed in *Arabidopsis thaliana* (L.) Heynh. protoplasts. eGFP fluorescence signal was observed by Olympus confocal microscope. Scale bars = 10 µm.

To explore the subcellular localization of StMYB proteins, the representative proteins StMYB6, StMYB19, and enhanced green fluorescent protein (eGFP) were constructed in the CAMV35S promoter, and then transferred into the *Arabidopsis thaliana* (L.) Heynh. protoplasts. The CAMV35S driven eGFP was transferred into *Arabidopsis thaliana* (L.) Heynh. protoplasts as a control (Figure 9). It is found that eGFP protein was distributed in different subcellular locations (Figure 9). However, the fusion proteins of StMYB6 with eGFP and StMYB19 with eGFP were mainly distributed in the nucleus (Figure 9).

## 3. Discussion

### 3.1. Evolutionary Analysis of the StMYB Gene Family

The MYB gene family is one of the largest transcription factor families in plants [5]. *MYB* genes play key roles in plant growth and development and a large number of biological processes [31,32,33,34]. With the continuous publication of genome data, the *MYB* gene family has been identified in many plants at the whole-genome level, including *Manihot esculenta* Crantz (299 members) [35], *Solanum lycopersicum* (121 members) [36], *Brassica napus* L. (249 members) [37], *Oryza sativa* L. (155 members) [23], *Arabidopsis thaliana* (L.) Heynh. (197 members) [23], soybean (244 members) [38], and so on. The genome size of *Manihot esculenta* Crantz is 742 MB [39], the *Solanum lycopersicum* genome size is 828.349 MB [40], the *Brassica napus* L. genome size is 912.169 MB [41], the rice genome size is 466 Mb [42], the *Arabidopsis thaliana* (L.) Heynh. genome size is 125 MB [43], and the soybean genome size is 1.025 GB [44]. The number of *MYB* genes identified in these plants is different, and their genome size is also different. By comparing the genome size of these plants and the number of identified *MYB* genes, it is found that the number of *MYB* genes in each plant did not depend entirely on the genome size. For example, the genome size of *Solanum lycopersicum* was medium, but the number of *MYB* genes in *Solanum lycopersicum* was the lowest.

Gene duplication events are the main reason for the expansion and evolution of plant genomes, and they are also the main factors promoting the expansion of gene families. The mechanisms of unequal crossing-over, duplication-dependent chromosome breakage, and nonhomologous exchange all lead to duplication events. Li et al. identified 8 pairs of tandem-duplicated *StMYB* genes and 31 pairs of segmental-duplicated *StMYB* genes from 230 *StMYB* genes [30]. In this study, 36 pairs of tandem-duplicated *StMYB* and 41 pairs of segmental-duplicated *StMYB* genes were identified from 158 *StMYB* genes (Figure 3). The different number of tandem duplication gene pairs we identified was because of the different identification criteria. We defined adjacent genes within 200 kb as tandem duplication gene pairs [45], while Li et al. defined adjacent genes with more than 70% identity in 200 kb as tandem duplication genes [30]. Similarly, apple, pear, and grape also experienced duplication events that led to the expansion of the MYB gene family in their genomes, suggesting that tandem duplication and segmental duplication together have promoted the expansion of the MYB gene family in different plants during the evolutionary process [13,24,46,47]. Duplicated genes may undergo different selection processes: achieving non-functionalization through silencing, achieving neofunctionalization by acquiring new functions, or being subfunctionalized by dividing the original functions of ancestral genes [48,49,50]. In this study, the expression levels of the identified tandem-duplicated genes were different in tissues (Figure 7). For example, *StMYB3* and *StMYB4* were a pair of tandem-duplicated genes, *StMYB4* was not expressed, but *StMYB3* was expressed in different tissues (Figure 3, Figure 7). We speculate that the reason may be that *StMYB4* lost the function of the original genes during evolution.

To further explain the evolutionary relationship of *MYB* genes in potato, we constructed a phylogenetic tree including potato, *Arabidopsis thaliana* (L.) Heynh., *Manihot esculenta* Crantz, *Solanum lycopersicum*, *Vitis vinifera* L., and *Oryza sativa* L. At the same time, we analyzed the motif compositions of *MYB* genes in these plants (Figure 5). From Figure 5, we can see that most of the *MYB* genes in all plants contained motif 1, motif 2, motif 4, and motif 5; although the motif composition of each subgroup was different, the motif composition within the same subgroup was similar. The motif compositions of the closely related members of the phylogenetic tree were consistent, which was also reported for *Phyllostachys edulis*, indicating that the MYB family is highly conserved between different plants [51]. Distinguished with Li et al.’s research [30], we analyzed the motif composition of *MYB* genes in different species, which can explore the conservativeness of *MYB* genes in different species and lay a foundation for syntenic analysis. By comparing the syntenic analysis of *MYB* genes in potato and other plants, we found that *StMYB* genes had the most homologous gene pairs with *MYB* genes in *Solanum lycopersicum* and *Manihot esculenta* Crantz, followed by *Vitis vinifera* L, which was consistent with Li et al. [30]. These results suggest that *MYB* genes in different plants may come from a common ancestor.

### 3.2. StMYB Genes Play Crucial Roles in Potato Growth and Development and Response to Phytohormone and Abiotic Stresses

The high-temperature, drought, and abiotic stresses seriously affected the yield of potato [52,53]. Secondary metabolites in plants, including flavonoids and anthocyanins, can help plants adapt to changing environments. Therefore, it is necessary to improve the resistance of the potato by regulating the genes related to the secondary metabolites, thereby increasing the yield. *MYB* genes can regulate the development of trichomes and root hairs [54,55], and they play a vital role in regulating the synthesis of plant secondary metabolites, such as flavonoids and anthocyanins [17,56,57,58], as well as helping plants resist abiotic stress [59,60,61]. To screen the *StMYB* genes that potentially regulate growth, development, and secondary metabolic synthesis, this study selected genes from each subgroup and measured their expression levels in different tissues. Different from our study, Li et al. focused on the expression levels of genes in SGs 14 and 20 in different tissues [30]. *AtMYB11*, *AtMYB12*, and *AtMYB111* were members of MYB SG 7 in *Arabidopsis thaliana* (L.) Heynh., and regulate flavonoid biosynthesis in all tissues [17]. *StMYB6* also belonged to SG 7, which is a homologous gene of *AtMYB11*, *AtMYB12*, and *AtMYB111*. qRT-PCR showed that it was expressed in all tissues of potato, but the highest expression level was found in flowers (Figure 7). In the future, we can verify whether *StMYB6* can regulate flavonoid synthesis through more in-depth experiments. *AtMYB* genes from SG 4 are transcriptional repressors of different branches of the phenylalanine metabolic pathway [62,63]. It has been reported that *AtMYB4* negatively regulates the formation of sinapate ester in the absence of ultraviolet radiation. When exposed to UV-B, the expression level of *AtMYB4* decreases, which increases the content of sinapate ester and plays a role in ultraviolet protection [62]. *AtMYB7* negatively regulates the synthesis of flavonoids and is a newly discovered transcriptional inhibitor involved in ultraviolet protection [64]. *StMYB89* and *StMYB12* also belonged to SG 4, and they were expressed in all tissues of potato, but were highest in mature flowers (Figure 1, Figure 4). Under phytohormone and abiotic stresses, their expression levels were also very high (Figure 8). *StMYB12* was the most responsive to GA, while *StMYB89* was the most responsive to IAA (Figure 8). *StMYB89* and *StMYB12* may play a crucial role in the growth and development of potato and resistance to adverse external environments. They can be used as candidate genes to improve potato quality, and their specific functions will be determined in future studies.

The expression of *MYB* genes is mainly regulated by a variety of hormones and abiotic stresses, including ABA, gibberellin, salt, drought, and high temperature. *AtMYB60* and *AtMYB96* from SG 1 can regulate stomatal movement and participate in drought stress through the ABA signaling pathway [65,66,67]. *AtMYB33* and *AtMYB101* from SG 18 and *AtMYB13* and *AtMYB15* from SG 2 respond to ABA expression [68,69]. *AtMYB2* from SG 20 is induced by ABA to regulate salt stress, and *AtMYB108* is involved in biotic and abiotic stresses [59,70]. *AtMYB44* from SG 22 regulates ABA-mediated stomatal closure under abiotic stress, and *AtMYB70*, *AtMYB73*, and *AtMYB77* in this SG are also involved in stress responses [71]. In order to explore *StMYB* genes involved in hormonal and abiotic stresses, we focused on the expression levels of *StMYB* genes from SG 1, SG 2, SG 18, SG 20, and SG 22 under different stress treatments, while Li et al. only focused on the expression patterns of genes of SG 1, SG 2, and SG 22 under stresses [30]. Using qRT-PCR, we found that *StMYB44* of SG 1, *StMYB27* of SG 2, *StMYB1* of SG 18, *StMYB3* and *StMYB53* of SG 20, and *StMYB19* of SG 22, which were homologous to stress responses genes, were expressed in all tissues. This indicated that they may play important roles in the potato growth and development. We predicted cis-acting elements in these genes promoters and found that their promoter regions contained multiple phytohormone responsiveness elements, and some *StMYB* genes contained the same copies of cis-acting elements, which may enhance the transcriptional regulation of genes and enable plants to adapt to changes in the environment (Figure 2D). To further identify candidate genes involved in hormone signaling pathways or abiotic stress responses, we measured their expression levels under hormone, salt, drought, and high-temperature treatments. The results showed that these genes were expressed under hormone and abiotic stresses, but the expression pattern of each gene was different (Figure 8). *StMYB44* had the highest expression under ABA treatment, which was consistent with the prediction of the composition of cis-acting elements in its promoter (Figure 2D, Figure 8). We can verify whether *StMYB44* performs the same functions as *AtMYB60* and *AtMYB96* in the future [65,66]. Except for the low expression level of *StMYB27* under high-temperature treatment, the expression level of *StMYB27* was high under all treatments, especially under the treatment of GA, which was consistent with the prediction of its promoter cis-acting elements (Figure 2D, Figure 8). *StMYB3*, *StMYB1,* and *StMYB53* were expressed under different treatments; *StMYB3* was the most responsive to ABA treatment, and *StMYB1* and *StMYB53* were the most responsive to high-temperature treatment (Figure 8). Their specific functions need to be further verified in the future. *StMYB19* was a gene that responded to hormone and abiotic stresses with high expression, which is consistent with the cis-acting element in its promoter (Figure 2D, Figure 8). We speculate that *StMYB19* may also be a gene that performs functions in the abiotic stress response, but the mechanism of its executive function is still unclear, which needs to be further verified in future experiments. Some *StMYB* genes screened in this study were potentially involved in ABA, GA, high temperature, and other abiotic stresses, while some *StMYB* genes screened by Li et al. were involved in salt tolerance [30].

In conclusion, a total of 158 *StMYB* genes were identified from potato genome. The gene structure, motif composition, chromosome localization, evolutionary relationship, expression patterns in different tissues, and response to hormone and abiotic stresses of *StMYB* genes were comprehensively analyzed. We have identified several potential candidate genes that regulate the synthesis of potato flavonoids or participate in hormones and stress responses. Our research lays a foundation for the future investigation of the potential functions of *StMYB* genes in the growth and development of potato.

## 4. Materials and Methods

### 4.1. Plant Materials and Growth Conditions

Potato Cultivar chuanyu 117 were used in this study. The potato plants were grown in a greenhouse at the College of Life Sciences, Sichuan Agricultural University, China. Different plant tissues were collected at different times after potato germination. Young buds were collected two weeks after germination. Roots, stems, leaves, and flowers were collected at the flowering stage. Stolons and young tubers were collected 15 days after anthesis. Mature tubers were collected approximately three months after potato germination. All samples were collected from three plants with similar growth. After the samples were collected, they were immediately put in liquid nitrogen and then put at −80 °C for storage.

The potatoes used for hormone and abiotic stresses were grown in MS medium with a pH of 5.8, 3% sucrose, and 1.5% agar in an external environment of 23 °C, with a photoperiod of 16 h of light and 8 h of darkness. Potato plantlets at four weeks were subjected to hormone, heat, drought, and salt stresses. For hormone treatment, potato plantlets were treated with 50 µM ABA, 10 µM IAA, and 50 µM GA3 for 24 h. For temperature stress, plantlets were exposed to 35 °C. For drought treatment, plantlets were treated with 260 µM mannitol for 24 h. For salt treatment, potato plantlets were treated with 150 mM NaCl for 24 h. Three plants with similar growth were selected for each stress treatment. After 24 h of different stress treatments, the third leaf from the top of the plant was collected. The collected samples were stored at −80 °C.

### 4.2. Identification of the StMYB Genes in Potato

We downloaded the potato genome sequence from the Potato Genome Sequencing Consortium (PGSC, http://potato.plantbiology.msu.edu/). We obtained the hidden Markov model (HMM) profile of the MYB domain (PF00249) through the Pfam protein family database (http://pfam.sanger.ac.uk/). The specific identification method of *MYB* genes was as follows. We downloaded the amino acid sequences of all *MYB* genes in *Arabidopsis thaliana* (L.) Heynh. from the Arabidopsis Information Resource (TAIR) library. All *MYB* genes were identified from the potato genome using downloaded *AtMYB* gene sequences as target sequences. The conserved domains of the potato *MYB* genes identified above were analyzed, and the genes that did not contain the PF00249 conserved domain were removed. Then, the identified potato *MYB* genes were run against BLASTp at National Center for Biotechnology Information (NCBI) to determine whether they belonged to the MYB gene family. Finally, we identified 158 *StMYB* genes from the potato genome. Basic information about these genes, including PIs, MWs, and subcellular localization, was predicted through the ExPASy website (https://web.expasy.org/protparam/).

### 4.3. Phylogenetics, Intron–Exon Structure, Motif Composition, and Cis-Acting Elements

We downloaded MYB protein sequences (*Arabidopsis thaliana* (L.) Heynh., *Solanum lycopersicum, Vitis vinifera* L., *Manihot esculenta* Crantz, and *Oryza sativa* L.) from the UniProt database (https://www.uniprot.org/) for constructing a multispecies maximum likelihood (ML) phylogenetic tree. Multiple amino acid sequences of MYB proteins from different plants were aligned using MUSCLE [72]. The ML phylogenetic tree including potato, *Arabidopsis thaliana* (L.) Heynh., *Solanum lycopersicum, Vitis vinifera* L., *Manihot esculenta* Crantz, and *Oryza sativa* L. was constructed with Mega 7.0 [73], and the specific parameters were the JTT + G + F model and 1000 bootstrap replications. The ML phylogenetic tree of potato and *Arabidopsis thaliana* (L.) Heynh. was constructed by the same method mentioned above, and the MYB protein sequences of potato and *Arabidopsis thaliana* (L.) Heynh. were aligned using MUSCLE before constructing the phylogenetic tree. The DNA and cDNA sequences of *MYB* genes were compared by online Gene Structure Display Service (http://gsds.cbi.pku.edu.cn/) to predict the structure of introns and exons. The conserved motifs of MYB proteins were determined by the Multiple Em for Motif Elicitation (MEME) online program (http://meme-suite.org/tools/meme) (Table S2). PlantCARE software (http://bioinformatics.psb.ugent.be/webtools/plantcare/html/?tdsourcetag=s_pcqq_aiomsg) was used to predict the cis-acting elements within 2000 bp upstream of all *StMYB* genes.

### 4.4. Chromosomal Mapping, Gene Duplication, and Synteny with Other Plants

We obtained *StMYB* gene localization information from circos and observed that all *StMYB* genes were located on different chromosomes of potato [74]. *StMYB* gene duplication events were analyzed with a multiple collinear scanning toolkit (MCScanX) [75]. The syntenic relationship between the *StMYB* genes and *MYB* genes from *Solanum lycopersicum, Manihot esculenta* Crantz, *Vitis vinifera* L., *Fagopyrum tataricum* Gaertn., *Arabidopsis thaliana* (L.) Heynh., *Nicotiana attenuate*, and *Oryza sativa* L. were determined using Dual Synteny Plotter software [76].

### 4.5. Expression Analysis of the StMYB Genes by qRT-PCR

Total RNA was extracted from the samples using the RNAout Kit (TIANGEN, China), and cDNA was synthesized using the PrimeScript™ 1st Strand cDNA Synthesis Kit (Takara, Japan) according to the manufacturer’s instructions. The expression levels of *StMYB* genes identified in the roots, stems, leaves, flowers, shoots, stolons, young tubers, and mature tubers were measured by qRT-PCR. Meanwhile, the expression levels of *StMYB* genes in potatoes treated with ABA, IAA, GA3, NaCl, mannitol, and heat were also measured. The qRT-PCR primers of the *StMYB* genes listed in Table S4 were obtained by online software primer 3 (https://www.ncbi.nlm.nih.gov/tools/primer-blast/). The qRT-PCR used elongation factor 1-α (ef1α) as internal reference gene [77]. The correlative expression data were calculated based on the 2^-△△CT^ method [78]. Three biological replicates were performed in the qRT-PCR experiment, and three technical replicates were performed in each biological replicate.

### 4.6. Subcellular Localization

The coding regions of *StMYB6* and *StMYB19* were amplified by PCR and fused to the pCAMBIA2300-eGFP plant expression vector. *Arabidopsis thaliana* (L.) Heynh. protoplasts were prepared from leaves of *Arabidopsis thaliana* (L.) Heynh. eGFP, StMYB6-eGFP, and StMYB19-eGFP were transferred into *Arabidopsis thaliana* (L.) Heynh. protoplasts. After 12 h transformation, the eGFP fluorescence was observed under an Olympus confocal microscope.

### 4.7. Statistical Analysis

We processed and analyzed our experimental data with the Origin Pro 2018b (OriginLab Corporation, Northampton, Massachusetts, USA), and we used the least significant difference (LSD) test to compare the data.

## Supplementary Materials

Supplementary materials can be found at https://www.mdpi.com/1422-0067/20/19/4847/s1.

## Abbreviations

ABA	abscisic acid
GA3	gibberellin acid 3
GFP	green fluorescent protein
HTH	helix–turn–helix
IAA	indole-3-acetic acid
MEME	Multiple Em for Motif Elicitation
MWs	molecular weights
NCBI	National Center for Biotechnology Information
PIs	isoelectric points
qRT-PCR	quantitative real-time polymerase chain reaction
R	repeat
SA	salicylic acid
SGs	subgroups
*StMYB*	*Solanum tuberosum* L. *MYB*
TFs	transcription factors
